# Contribution of the Transcription Factors Sp1/Sp3 and AP-1 to *Clusterin* Gene Expression during Corneal Wound Healing of Tissue-Engineered Human Corneas

**DOI:** 10.3390/ijms222212426

**Published:** 2021-11-17

**Authors:** Christelle Gross, Gaëtan Le-Bel, Pascale Desjardins, Manel Benhassine, Lucie Germain, Sylvain L. Guérin

**Affiliations:** 1 Centre Universitaire d’Ophtalmologie-Recherche (CUO-Recherche), Centre de Recherche du CHU de Québec, Axe Médecine Régénératrice, Hôpital du Saint-Sacrement, Québec, QC G1S 4L8, Canada; christelle.gross@crchudequebec.ulaval.ca (C.G.); gaetan.lebel17@gmail.com (G.L.-B.); Pascale.desjardins.1@ulaval.ca (P.D.); benhassine.manel@hotmail.fr (M.B.); Lucie.germain@fmed.ulaval.ca (L.G.); 2 Centre de Recherche en Organogénèse Expérimentale de l’Université Laval/LOEX, Génie Tissulaire et Régénération, Centre de Recherche du CHU de Québec, Axe Médecine Régénératrice, Québec, QC G1V 0A6, Canada; 3 Département d’Ophtalmologie, Faculté de Médecine, Université Laval, Québec, QC G1V 0A6, Canada; 4 Département de Chirurgie, Faculté de Médecine, Université Laval, Québec, QC G1V 0A6, Canada

**Keywords:** *clusterin*, human cornea, tissue-engineering, 3D corneal model, wound healing, transcription factor, promoter, Activator Protein 1 (AP1), Specific protein 1 and 3 (Sp1/Sp3)

## Abstract

In order to reduce the need for donor corneas, understanding of corneal wound healing and development of an entirely tissue-engineered human cornea (hTECs) is of prime importance. In this study, we exploited the hTEC to determine how deep wound healing affects the transcriptional pattern of corneal epithelial cells through microarray analyses. We demonstrated that the gene encoding *clusterin* (*CLU*) has its expression dramatically repressed during closure of hTEC wounds. Western blot analyses confirmed a strong reduction in the expression of the *clusterin* isoforms after corneal damage and suggest that repression of *CLU* gene expression might be a prerequisite to hTEC wound closure. Transfection with segments from the human *CLU* gene promoter revealed the presence of three regulatory regions: a basal promoter and two more distal negative regulatory regions. The basal promoter bears DNA binding sites for very potent transcription factors (TFs): Activator Protein-1 (AP-1) and Specificity protein-1 and 3 (Sp1/Sp3). By exploiting electrophoretic mobility shift assays (EMSA), we demonstrated that AP-1 and Sp1/Sp3 have their DNA binding site overlapping with one another in the basal promoter of the *CLU* gene in hCECs. Interestingly, expression of both these TFs is reduced (at the protein level) during hTEC wound healing, thereby contributing to the extinction of *CLU* gene expression during that process. The results of this study contribute to a better understanding of the molecular mechanisms accounting for the repression of *CLU* gene expression during corneal wound healing.

## 1. Introduction

Visual acuity is directly correlated with the integrity of the anatomical structures of the eyes. One of these key components is the cornea, consisting of the outer layer of the eye. The cornea is a transparent structure, crucial for light refraction to the retina that accounts for three-quarters of the total eye refractive power. Hence, maintaining corneal transparency is of basic necessity; otherwise, visual acuity will be inevitably affected [1]. Moreover, its integrity is essential because it also constitutes the first barrier of the ocular surface against pathogen infections [1]. However, because of this specific localization, it is the first structure altered by thermic, chemical or mechanical damages. According to the first global report on vision launched in 2019 by the World Health Organization (WHO), at least 2.2 billion people worldwide suffer from a vision impairment or blindness. Of these, corneal opacities consecutive to corneal damages represent the fifth cause of visual deficiency with 4.2 million patients. In North America, the incidence of corneal wounds accounts for 37% of all visual deficiency and 23% of ocular consultation, which makes it a large proportion of all visual disabilities [2].

Corneal repair and regeneration mainly depend on the limbal stem cells located at the junction between the corneal and conjunctival epithelium. Indeed, a compromised limbal niche is a key factor of the injury’s severity, the prognostic of healing being poor when more than 50% of the limbal area is damaged [3]. Furthermore, the loss of stem cells can lead to the limbal stem cell deficiency syndrome that also causes a partial or complete opacification of the cornea [4]. Corneal barrier rupture can also frequently cause infection [5]. If not treated rapidly and properly, pathogens can penetrate into the cornea and cause damages to the subjacent tissues [6]. The consequences of such corneal damages are dramatic and can lead to the complete loss of vision [7]. According to the severity of the trauma, actual treatments, which aim to improve epithelial healing and/or reduce inflammation, may not be satisfactory. In some cases, a corneal transplantation or eye enucleation may even be required [8]. However, the growing popularity of refractive surgeries renders donor corneas unusable, thus reducing the number of available grafts. In order to reduce the need for donor corneas, understanding of corneal wound healing and development of an entirely tissue-engineered human cornea (hTECs) is of prime importance. We succeeded in producing two-layer hTECs (epithelium and stroma) made up of primary cultured cells grown on a naturally secreted extracellular matrix that show characteristics very similar to those of the native cornea, including the expression of the epithelial barrier marker ZO-1, the differentiation marker keratins K3/K12, the corneal integrins αvβ6 and α2β1 and integrin subunits β4, α3 and α6 and the subepithelial basement membrane and stromal components laminin V, collagen types I, IV, V and VII, to name a few [9,10,11,12,13]. Over the last 20 years, we used this substitute to study the mechanistic of wound healing [2,9,11,14,15,16,17,18]. The dynamic of wound closure was found to be very similar between the hTEC and the native cornea [11]. Because of these similarities, the hTEC represents an outstanding model that we can exploit to study in detail the cellular and molecular mechanisms of corneal wound healing.

Corneal wound healing is a complex event involving many processes, such as cell death, proliferation, migration, adhesion and differentiation [19]. During each of these steps, genes and enzymes expression are altered to allow proper wound closure [11]. In this context, clusterin (CLU), an extracellular chaperone [20], is a target of interest, as it is involved in multiple physiological processes including apoptotic cell death [21,22], cell adhesion [23], migration [24] and proliferation [25] and tissue remodeling [26], to name a few. CLU-overexpression is associated with different pathologic contexts (aging, cancer tumorigenesis and chemoresistance, neurodegeneration, cardiovascular diseases) including eye pathologies (Fuch’s Dystrophies, age-related macular degeneration and amyloid plaques of corneal dystrophy) [27,28,29,30,31,32,33]. However, while the CLU-mediated signaling pathways in the eye [34] are beginning to be clarified [33,35,36], the molecular basis of its gene expression remains unclear. Few reports identified multiple binding sites for a variety of transcription factors (TFs) along the *CLU* gene promoter, including activator Protein 1 (AP-1), Specificity Protein 1 (Sp1), Nuclear Factor 1 (NFI), Signal Transducers and Activators of Transcription (STAT), MYCN Proto-oncogene and Heat Shock Factor (HSF), to name a few [37,38]. Although a handful of them reported the characterization of the regulatory sequences that are critical to ensure proper transcription of the CLU gene [39,40,41,42,43,44,45,46], none have ever investigated their contribution to human wound healing.

Human *CLU* is a gene located on the reverse strand of chromosome 8. The *CLU* gene is organized in nine exons and generates a transcript of approximatively 2 kb [47]. It is ubiquitously expressed and encodes a glycosylated and secreted protein of approximatively 75–80 kDa (sCLU). The human *CLU* gene encodes three main transcripts: isoform 1, which is the most abundant transcript variant (accession number GenBank NM_001831.2); isoform 2 (accession number GenBank NR_038335); and isoform 3 (accession number GenBank NR_045494.1) [37,48]. All variants are composed of a unique exon 1 that probably originates from two alternative starting sites, and share the remaining exons 2–9 sequence [37]. In the early stages of maturation, *CLU* mRNA is translated into an unfolded precursor protein. Then, the precursor is directed to the endoplasmic reticulum and Golgi apparatus, where it is subjected to glycosylation and proteolytic cleavage into α and β subunits (of 40 kDa each and called cCLU). The α and β chains are reassembled by disulfide bonds, leading to a mature heterodimeric protein of 75–80 kDa (sCLU) [49]. More recently, an alternative messenger RNA was found [50]. Authors have identified a new form of CLU isoform 1 lacking Exon 2 that results in the production of a shorter non-glycosylated and non-secreted 55–60 kDa CLU protein (nuclear-nCLU). CLU has been associated to contradictory cellular functions, such as cell survival and apoptosis. These apparently ambiguous functions appear to be attributed to both sCLU, which has pro-survival and tumorigenic functions, and nCLU, which exhibits a pro-apoptotic activity [28,50,51].

In the present study, we exploited the hTEC as a model to better understand the molecular mechanisms regulating *CLU* gene expression during corneal wound healing. We demonstrated that *CLU* gene expression was severely repressed during hTEC wound healing and that both positive and negative regulatory elements that contribute to its transcription in hCECs are present in both the basal promoter and 5′-flanking sequence of the *CLU* gene. Furthermore, both the positive, ubiquitous TFs Sp1 and Sp3 (Sp1/Sp3), and AP-1, whose protein expression was also found to decrease in wounded hTECs (with the exception of Sp3), were shown to contribute to *CLU* gene transcription by interacting within its basal promoter in vitro.

## 2. Results

### 2.1. hTECs Wound Healing Alters CLU Gene Expression in Human Corneal Epithelial Cells

**Figure 1:**
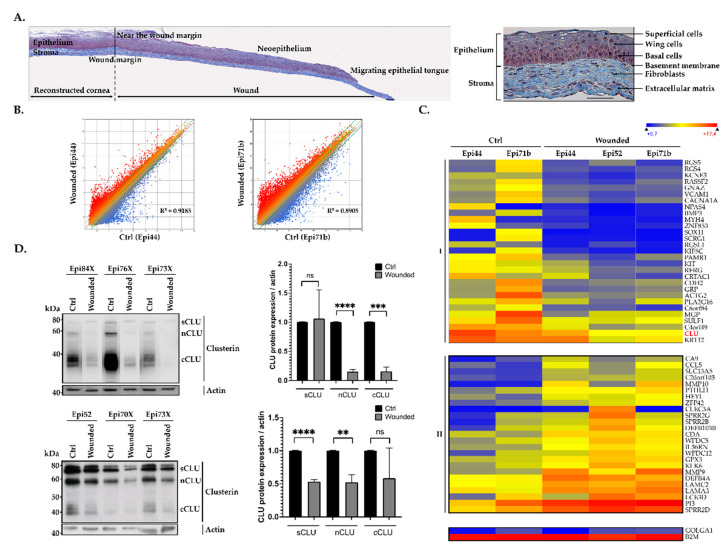
Gene expression patterns in response to corneal damage. (**A**) Composite images showing a complete histological (Masson’s Trichrome staining cells are pink and collagen is blue) view of wounded HTECs (with Epi52) at 4 days following corneal damage (left panel) and unwounded hTECs as a control (right panel). The wound margin created by the biopsy punch and the various elements which composed hTECs are indicated. Scale bar: 50 μm. (**B**) Scatter plot of log2 of signal intensity from 60,000 different targets covering the entire human transcriptome of cells isolated from the central area of wounded hTECs (in the *y-axis*) plotted against cells from unwounded hTECs (in the *x-axis*) and assembled using either Epi44 (left panel) or Epi71b (right panel) hCECs. (**C**) Heatmap representation of the 54 most deregulated genes expressed by centrally wounded relative to unwounded (negative control) hTECs. The color scale used to display the log2 expression level values is determined by the Hierarchical clustering algorithm of the Euclidian metric distance between genes. Genes indicated in dark blue correspond to those whose expression is very low, whereas highly expressed genes are shown in orange/red. Microarray data for the housekeeping genes β2-microglobulin (*B2M*) and golgin subfamily A member 1 (*GOLGA1*) that are expressed respectively to very high and low levels in all types of cells are also shown. (**D**) Western blot (left panel) and quantification analysis (right panel) of CLU protein isoforms either in hTECs (top panel) or in human epithelial corneal cells (bottom panel). Actin is also shown as an internal control. **: Values considered to be statistically significant from those obtained with the control (Ctrl) protein extract (*p* value < 0.001). ***: Values statistically significant from those obtained with the control (Ctrl) protein extract (*p* value < 0.001). ****: Values statistically significant from those obtained with the control (Ctrl) protein extract (*p* value < 0.0001). ns: Values considered to be statistically non-significant.

Biopsies from central areas of both wounded and unwounded (used as negative controls) hTECs were used to extract total RNA in order to conduct gene profiling analyses on microarrays. A scatter plot analysis of the 60,000 different transcripts contained on the arrays provided evidence that hCECs from the central area of wounded hTECs have a distinctive pattern of expressed genes from that yielded by unwounded hTECs, as revealed by the dispersion of the normalized signals that appear as a cloud of dots in Figure 1A, and the slope of the regression curve (R^2^ = 0.9185 for Epi 44 and R^2^ = 0.8905 for Epi 71b). Analysis for all the genes showing a two-fold or more expression variation unique to hCECs from the central areas of wounded corneas paired with those from unwounded hTECs was then generated. A total of 2754 genes fitted into that category of differentially regulated genes when hCECs from the central area are compared between wounded and unwounded hTECs. We next examined the data files from the microarrays to sort out genes whose expression was the most deregulated in hCECs between the central areas of the wounded corneas and their relative unwounded substitutes. The 54 most deregulated genes were classified according to their variation during wound healing and arbitrarily named class I and II. As shown in Figure 1B, expression of the genes identified as class I was dramatically reduced in the central wound (Wounded) compared to their normal level in the unwounded hTECs (Ctrl). On the other hand, genes from class II seek their expression strongly increased in the central wound.

Among the 54 genes identified above in Figure 1B, that encoding clusterin (*CLU*) was among the most strongly expressed in control hTECs, whereas its expression is dramatically reduced in the central area of wounded hTECs. Indeed, the *CLU* average miroarray linear signals drops from 18,624 in unwounded hTECs to only 1146 in wounded hTECs (corresponding to a 16-fold repression). Consistent with these results, CLU is known to be involved in multiple physiological mechanisms linked to wound healing processes (including apoptosis, cell adhesion, migration and proliferation, tissue remodeling, etc.) [21,22,23,24,25,26]. We then examined whether the repression in *CLU* gene expression observed in microarray also translates into a corresponding reduction at the protein level. As shown in Figure 1C, the variations in *CLU* gene expression observed in the microarray were also validated at the protein level by western blot analyses. All CLU isoforms (sCLU, cCLU and nCLU protein) were expressed in hTECs and hCECs prior to wounding (top and bottom panels, respectively). However, expression of both nCLU and cCLU was dramatically reduced in the wounded hTECs, whereas the reduction was less important in hCECs. No change in sCLU expression could be observed in the hTEC, whereas it was reduced by 50% in hCECs.

### 2.2. CLU Gene Transcription Is Modulated by Both Positive and Negative Regulatory Elements

**Figure 2:**
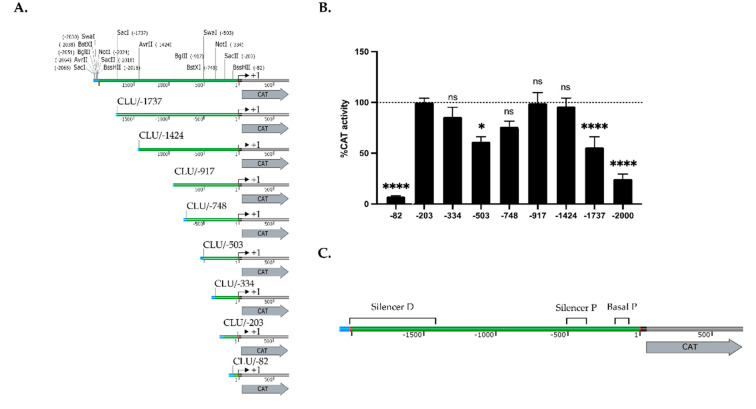
Transfection of the *CLU* gene promoter in human corneal epithelial cells. (**A**) Representation of the CLU/CAT recombinant plasmids used for transfection analyses. Numbers indicate the 5′ position relative to the *CLU* theoretical mRNA start site (indicated by a curved arrow). (**B**) CAT activities measured following transfection of the CLU/CAT constructs shown in panel (**A**) in human corneal epithelial cells. CAT activity is expressed relative to the level directed by CLU/-203 construct. Values considered to be statistically significant from those obtained with the CLU/-203 construct are indicated (* and ****: *p* values < 0.05 and < 0.0001, respectively). ns: Values considered to be statistically non-significant. (**C**) Schematic representation of the basal promoter (basal P) and both the proximal and distal silencers along the *CLU* promoter and 5′-flanking sequence, based on the transfection results from the panel (**B**).

In order to investigate further the mechanism by which *CLU* gene expression is regulated during wound healing, we cloned different DNA fragments from the human *CLU* gene promoter and 5′-flanking region upstream of the CAT reporter gene and transfected the resulting plasmid constructs into hCECs (Figure 2A).

The plasmid CLU/-203 that contains the *CLU* promoter sequence from 3′ position +1 to 5′ position -203 relative to the putative mRNA start site [37] yielded an easily detectable CAT activity upon transfection of HCECs (Figure 2B). However, deletion of the *CLU* promoter to position -82 (in plasmid CLU/-82) nearly abolished CAT activity relative to the level directed by the CLU/-203 construct (14-fold repression). In contrast, extending the *CLU* promoter by 131 bp to position -334 (in CLU/-334) had no influence.

Extenting further the *CLU* promoter to position -503 (in CLU/-503) resulted in a modest repression (1.6-fold repression) of the CAT activity compared to those of CLU/-203, which is gradually relieved upon transfection of the plasmid CLU/-748 (1.3-fold repression) and CLU/-917 (no repression). Again, extending further the *CLU* promoter to position -1424 (CLU/-1424) had no impact on CAT activity, whereas transfection of either CLU/-1737 or CLU/-2000 caused a progressive repression of CAT activity (1.8- and 4.1-fold repression, respectively). Collectively, these results indicate that transcription of the *CLU* gene is under the control of two negative silencer elements: a strong one, located between positions -1424/-2000 and refered to as the distal silencer (Silencer D; Figure 2C), and a weaker one, located between positions -334/-748 and designated as the proximal silencer (Silencer P; Figure 2C). They also suggest that regulatory elements required to ensure basal expression of the *CLU* gene are present between positions -82 and -203 (designated as the basal promoter (P).

### 2.3. Analysis of the Transcription Factors Binding to the CLU Basal Promoter in HCECs

**Figure 3:**
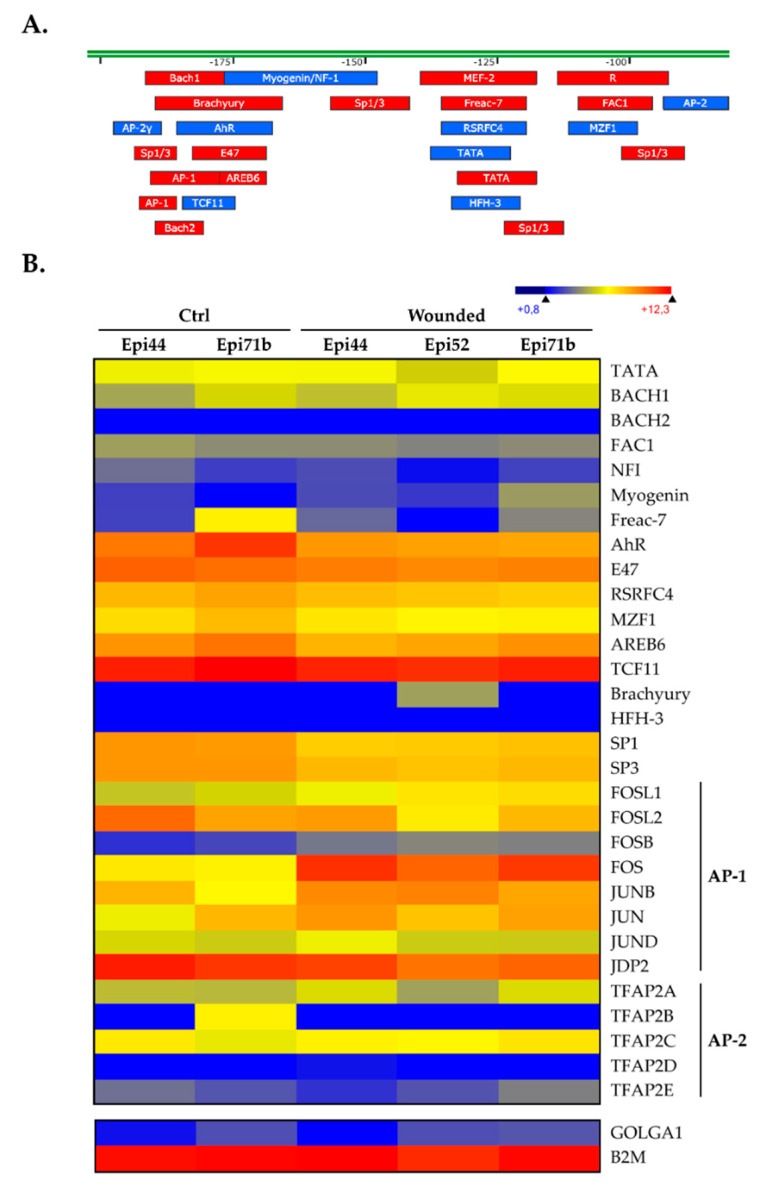
Putative transcription factor binding sites along the CLU promoter sequence. (**A**) Schematic representation of the human *CLU* promoter -82/-203 segment along which the presumed transcription factor binding sites. TF target sites identified on the DNA top strand are indicated in red, whereas those indicated in blue are located on the bottom strand. (**B**) Heatmap representation of the transcriptional profiles of all the TFs expressed by hTECs for which a putative target site was identified in panel (**A**). Microarray data for *B2M* and *GOLGA1* are also shown as controls.

We then subjected the 2 Kbp segment from the *CLU* gene upstream regulatory sequence to a search with the TFSEARCH program, a tool that can identify putative DNA target sequences for most transcription factors (TFs). To be consistent with the transfection results shown in Figure 2C, we focused our attention toward the -82/-203 regulatory element, as its presence is absolutely required to ensure basal *CLU* promoter function. Interestingly, a particularly high number of putative target sites for two TFs families were identified in this promoter region of only 131 pb: 4 sites for *Specificity protein1 and 3* (Sp1/Sp3), two closely related TFs that recognize the same target site in DNA, and one site for *Activator Protein-1* (AP-1) (Figure 3A). These two TFs families have been consistently reported to function as activators of gene expression [52,53,54]. We next examined the pattern of expression for these TFs in the hTECs by searching the microarray data files used for generating the data appearing in Figure 1. As shown in Figure 3B, all of these TFs (including the AP-1 constituting subunits c-Jun (JUNC), b-Jun (JUNB), d-Jun (JUND), c-Fos (FOSC), FOS-like 1 and 2 (FOSL1 and FOSL2) and FosB (FOSB)) are easily detectable in hTECs. A significant increase in the expression of many of them (FOSL1, FOSB, FOSC and JUNB) was observed in our wounded hTECs compared to the expression observed in the unwounded, control hTECs. Interestingly, only the TFs Sp1 and Sp3 had their transcription reduced in wounded hTECs.

### 2.4. Members from the AP1 and Sp1 Families Bind to the CLU Basal Promoter in hCECs

**Figure 4:**
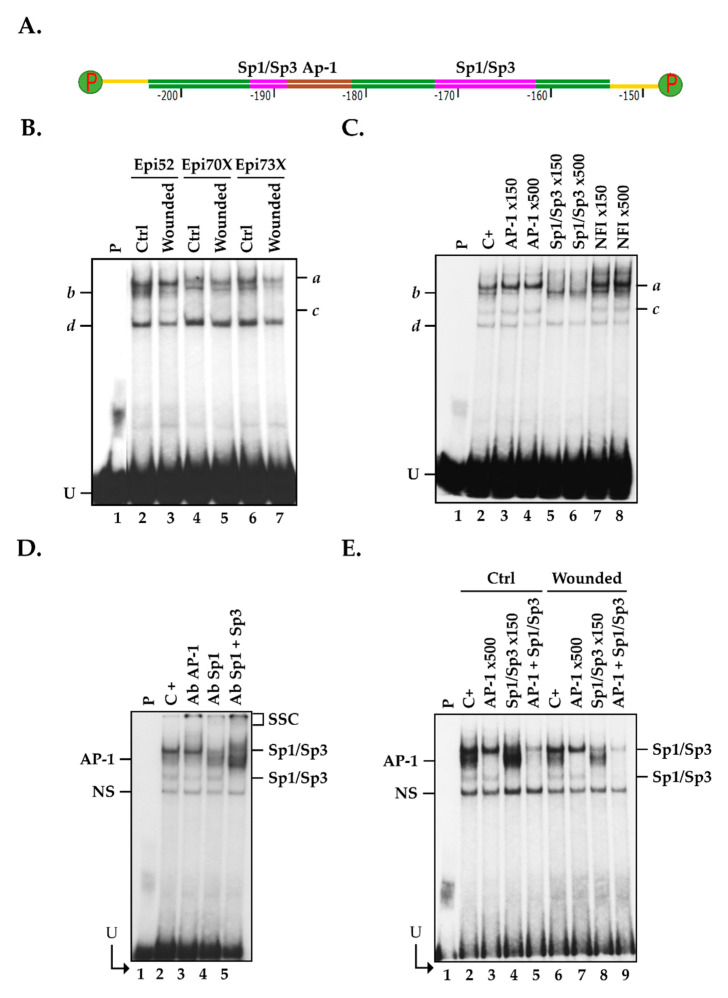
Expression and binding of the TFs AP-1 and Sp1/Sp3 to the *CLU* basal promoter region using nuclear extracts from hCECs. (**A**) Schematic representation of the 50 bp segment from the human *CLU* promoter (-153 to -203) used as a labeled probe in EMSA that also bears putative binding sites for AP-1 (from -188 to -182 (brown)) and Sp1/Sp3 (from positions -194 to -186 and -170 to -161 (pink)). (**B**) Nuclear proteins (15 µg) prepared from three different populations of hCECs (Epi52, Epi70X and Epi73X) were incubated with the CLU-labeled probe and formation of DNA-protein complexes monitored by EMSA. (**C**) Nuclear proteins from unwounded hCECs (Epi 70X) were incubated with the CLU-labeled probe either alone (C+) or in the presence of a 150- or 500-fold molar excess of unlabeled competitor oligomers bearing the high affinity recognition target site for AP-1, Sp1/Sp3 or NFI. (**D**) HCECs nuclear proteins (Epi70X) were incubated with the CLU-labeled probe either alone (negative control: C+) or with antibodies that can recognize the c-Fos or c-Jun AP-1 isoforms (Ab AP-1), or Sp1 and/or Sp3 prior to separation of the DNA-protein complexes by EMSA. SSC: supershifted complexes formed upon addition of the antibodies. (**E**) Nuclear proteins from unwounded (Ctrl) or scratch-wounded (Wounded) hCECs (Epi 52) were incubated with the CLU probe either alone (C+) or in the presence of either a 150- or 500-fold molar excess of unlabeled competitor oligomers bearing the target site for either AP-1 or Sp1/Sp3. The positions of both the AP-1 and Sp1/Sp3 DNA-protein complexes are indicated. P: labeled probe without added proteins; U: free probe; N.S.: non-specific complex.

The -82/-203 basal promoter segment bears DNA binding sites for very potent transcriptional activators: AP-1 and Sp1/Sp3 (identified along the *CLU* promoter in Figure 3A). To assess whether AP-1 and Sp1/Sp3 can indeed bind to the *CLU* promoter in vitro, nuclear extracts were prepared from three populations of hCECs (hCECs-52, hCECs-70X and hCECs-73X) and used in electrophoretic mobility shift assay (EMSA). To that purpose, we 5′end-labeled a 50 bp, double-stranded oligonucleotide bearing the *CLU* gene promoter sequence from positions -153 to -203 (CLU -203/-153) that also bears both the AP-1 and Sp1/Sp3 putative target sites (Figure 4A), and incubated this labeled probe with nuclear proteins from all populations of hCECs. Separation of the reaction mixtures on EMSA revealed the formation of four DNA-protein complexes on gel (*a* to *d*; Figure 4B). Although equal amounts of nuclear proteins (15 μg) were used for the assay, the extract isolated from wounded hCECs (Wounded) yielded weaker DNA-protein signals on gel (Figure 4B). Addition of either a 150- or 500-fold molar excess of an unlabeled, double-stranded oligonucleotide bearing the high affinity prototypical DNA binding site for AP-1 entirely eliminated formation of complex *b* (when a 500-fold excess is used), whereas a similar unlabeled oligomer bearing the high affinity binding site for Sp1/Sp3 entirely prevented that of complexes *a* and *c* (Figure 4C). On the other hand, a similar oligomer bearing the high affinity target site for the unrelated Nuclear Factor I (NFI) transcription factor did not alter the formation of any of these complexes, even when used at a 500-fold molar excess. These results demonstrate that the TFs AP-1 and Sp1/Sp3 are constituents of complex *b* and both complexes *a* and *c*, respectively. The significant reduction in the formation of both the AP-1 and Sp1/Sp3 complexes combined to the formation of new, slow-migrating supershifted complexes (SSC) upon addition of a polyclonal antibody that recognizes AP-1 (lane 3), Sp1 (lane 4), or the combination of both Sp1 and Sp3 (lane 5), provided evidence that formation of complexes *b* and *a/c* resulted from the recognition of the CLU-203/-/53 labeled probe by AP-1 and Sp1/Sp3, respectively (Figure 4D). Formation of complex *d* results from the recognition of the labeled probe by non-specific DNA binding proteins, as is often observed in EMSA [17,18,52,53,54,55,56,57].

We next examined whether formation of both the AP-1 and Sp1/Sp3 complexes was altered in scratch-wounded relative to unwounded hCECs grown as monoloyers. As shown in Figure 4E, addition of the unlabeled Sp1/Sp3 oligonucleotide indeed reduced binding of Sp1 but also caused an increased recognition of the labeled probe by AP-1 when nuclear proteins from unwounded hCECs were used (compare lane 2 with lane 4) but not those from scratch-wounded hCECs (compare lane 6 with lane 8). On the other hand, formation of the Sp1/Sp3 complexes was not affected when AP-1 was totally prevented from interacting with the labeled probe by the addition of the AP-1 unlabeled competitor, irrespective of the conditions used (wounded or unwounded hCECs; compare lane 2 with lane 3 and lane 6 with lane 7). Furthermore, incubation of nuclear extracts with both the unlabeled AP-1 and Sp1/Sp3 oligonucleotides almost entirely prevented formation of their respective DNA-protein complex in EMSA (compare lane 2 with lane 5, and lane 6 with lane 9). Interestingly, there is a marked decrease in AP-1 DNA binding when hCECs are wounded, relative to unwounded cells, irrespective of whether Sp1 is prevented from interacting with its target site (by competing with the unlabeled Sp1 oligomer; compare lanes 4 and 8) or not (compare lane 2 and 6), despite that identical amounts of nuclear proteins were used in EMSA. These results, therefore, suggest that Sp1/Sp3 competes with AP-1 for the recognition of the *CLU* gene promoter and that Sp1/Sp3 has a stronger binding affinity to its *CLU* target site than AP-1 does in HCECs.

### 2.5. Positioning of the Functional Sp1/Sp3 Binding Site in the CLU Promoter In Vitro

**Figure 5:**
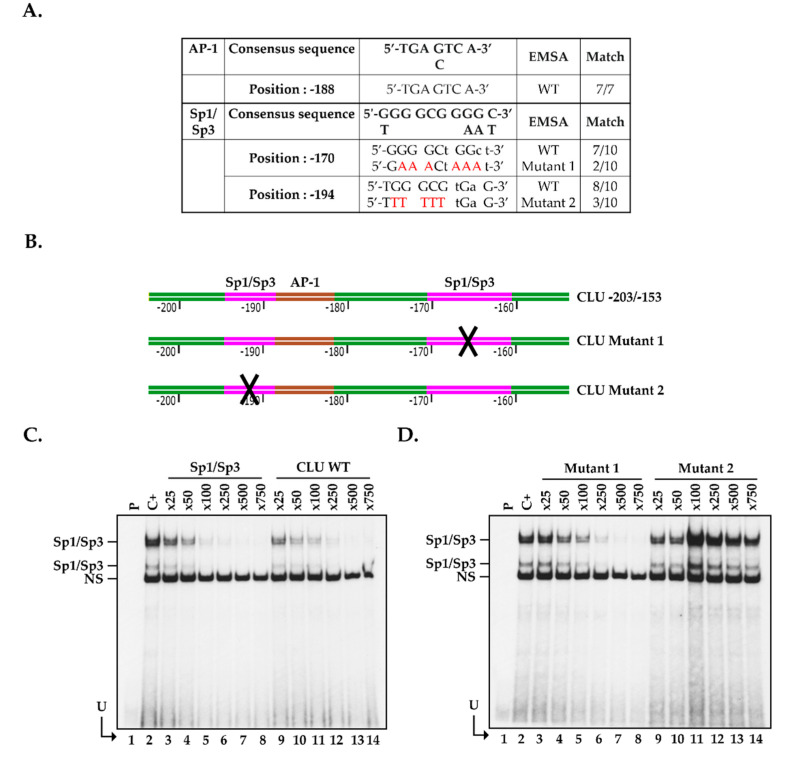
Sp1/Sp3 binding sites in the -203/-153 *CLU* promoter segment. (**A**) DNA sequence of the AP-1 and Sp1/Sp3 target sites from the *CLU* promoter identified at position -170, -188 and -194, respectively, aligned with their corresponding prototypical sequence. The nucleotides preserved between the *CLU* TF sites and their consensus sequences are shown in capital letters along with those selected for site-directed mutation (in red). (**B**) Schematic representation of the -203/-153 *CLU* promoter segment used in EMSA, and its derivatives that bear mutations into either the -170 (CLU Mutant 1) or the -194 (CLU Mutant 2) Sp1 site (the mutated site is indicated by an ‘X’). (**C**) Nuclear proteins (15 µg) from control hCECs (Epi 70x) were incubated with the Sp1/Sp3-labeled probe bearing the consensus sequence for the Sp1/Sp3 TFs either alone (C+) or in the presence of increasing molar excesses (25 to 750-fold) of unlabeled CLU-203/-153, or of competitors bearing the target site for Sp1/Sp3 and run at 110V for 6.5 h. P: labeled probe without added proteins, U: free probe. (**D**) Same as in panel (**C**), except that the Sp1/Sp3-labeled probe was incubated either alone (C+) or in the presence of the unlabeled CLU Mutant 1 or CLU Mutant 2 oligomers.

The -203/-153 segment from the *CLU* basal promoter bears two putative Sp1/Sp3 target sites (located at positions -194 and -170 relative to the *CLU* mRNA start site). The -194 Sp1/Sp3 binding site is, therefore, located very close to the AP-1 target site (located at position -188) and shares 8 nucleotides out of the 10 with those typically found in the prototypical Sp1/Sp3 target site (Figure 5A). In order to determine which of the -194 or -170 Sp1/Sp3 sequence indeed binds this TF, we generated double-stranded oligonucleotides in which most of the G residues from both the -194 and -170 Sp1/Sp3 target sites were replaced by Ts (Figure 5A,B) and used them as unlabeled competitors in EMSA. To that purpose, and to avoid any interference from AP-1, we 5′end-labeled a 25 pb oligomer bearing only the high affinity, Sp1/Sp3 prototypical target site (Supplementary Table S1) and used it as the labeled probe in EMSA. As shown in Figure 5C, incubation of this labeled probe with nuclear proteins from control hCECs yielded the formation of the typical Sp1/Sp3 DNA-protein complexes in EMSA (lane 2) [52,53,54,55,56,57]. As expected, the addition of increasing amounts (25- to 750-fold molar excesses; lanes 3 to 8) of the unlabeled high-affinity Sp1/Sp3 oligomer very efficiently competed for the formation of the Sp1/Sp3 complexes on gel, a 500-fold molar excess of the unlabeled competitor (lane 7) being sufficient to entirely prevent their formation. As shown in Figure 5C (lanes 9 to 14), competing with identical amounts of the CLU -203/-153 oligomer that bears both the CLU putative Sp1/Sp3 and AP-1 sites proved as efficient as the prototypical Sp1/Sp3 oligomer to compete for the formation of the Sp1/Sp3 complexes in EMSA. Interestingly, the addition of 50- to 750-fold molar excesses of the CLU oligomer that bears mutations in the -170 Sp1 site (CLU Mutant 1; Figure 5D, lanes 3 to 8) was also as effective, as both the high affinity Sp1 and CLU-203/-153 oligomers to compete for the formation of the Sp1/Sp3 complexes. On the other hand, the oligomer bearing the mutated -194 Sp1/Sp3 binding site was totally unable to compete for the formation of these complexes (CLU/Mutant 2; Figure 5D, lanes 9–14). These results clearly identify the -194/-185 segment of the *CLU* promoter as the preferred target site for Sp1/Sp3. They also demonstrate that AP-1 and Sp1/Sp3 share overlapping binding sites on the *CLU* gene basal promoter, which is consistent with the observations shown in Figure 4C.

### 2.6. Influence of Cell Damage on the Binding of Sp1/Sp3 to the CLU Promoter in hCECs

**Figure 6:**
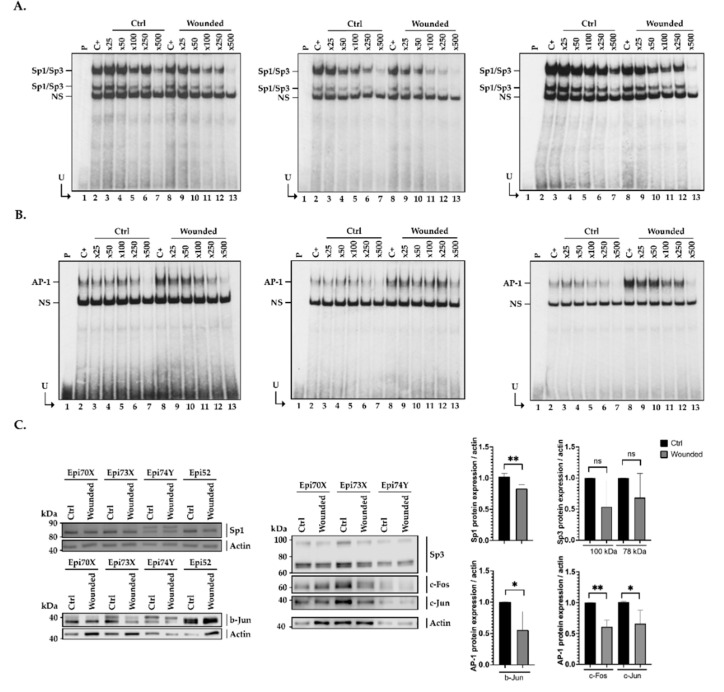
Sp1/3 and AP-1 binding after hCECs damages. (**A**) Nuclear protein (15 µg) from control (Ctrl) and damaged (Wounded) hCECs (Epi 70x) were incubated with either the Sp1/Sp3 (**panel A**) or the AP-1 (**panel B**) labeled probe bearing the consensus sequence for the TFs Sp1/Sp3 and AP-1 TFs, respectively, either alone (C+) or in the presence of increasing molar excess (25 to 750-fold) of unlabeled competitor oligonucleotides. P: labeled probe without added proteins, U: free probe. Unlabeled competitor oligonucleotides used in the two left panels (**A**,**B**) bears the specific target site for the TFs Sp1/Sp3 (**A**) or AP-1 (**B**). Central panels are the same as in left panels except that DNA-protein complexes were competed with unlabeled CLU -203/-153 while the complexes in the right panel are competing with CLU Mutant 2. (**C**) Western blot (left and middle panels) and quantification analysis (right panel) of the AP-1 (c-Fos, c-Jun and b-Jun isoforms) and Sp1/Sp3 TFs expression in hCECs nuclear extract (Epi52, Epi70X, Epi73X, Epi 74Y). Actin expression was monitored as a normalization control. *: Values considered to be statistically significant from those obtained with the control (Ctrl) protein extract (*p* value < 0.05). **: Values considered to be statistically significant from those obtained with the control (Ctrl) protein extract (*p* value < 0.01). ns: Values considered to be statistically non-significant.

Examination of Figure 4 revealed a weaker formation of the Sp1/Sp3, but especially of the AP-1 complex in wounded hCECs compared to the control condition when the CLU-203/-153 oligomer is used as the labeled probe in EMSA. We again exploited the EMSA to determine whether the Sp1/Sp3 affinity for its prototypical sequence is influenced by a context of injury. To achieve this goal, nuclear proteins isolated from control (Ctrl) or scratch-wounded (Wounded) hCECs were incubated with the Sp1/Sp3 or AP-1 high affinity labeled probes, either alone (C+) or in the presence of increasing amounts of unlabeled competitors (Sp1/Sp3, CLU-203/-153 or CLU/Mutant 2) and the formation of DNA-protein complexes monitored by EMSA. Incubation of nuclear proteins from control (Ctrl) and damaged (Wounded) hCECs with either the Sp1/Sp3 (Figure 6A) or AP-1-labeled probe (Figure 6B) yielded to the formation of the typical Sp1 and AP-1 complexes, respectively (lanes 2 and 8 of each gel). In both cases, the addition of increasing amounts of unlabeled TFs specific oligonucleotides (respectively Sp1/Sp3 in the left panel of Figure 6A and AP-1 in the left panel of Figure 6B) progressively prevented the formation of both TF DNA-protein complexes to reach almost complete disapearence when a 500-fold molar excess of competitor is used. Similarly, both Sp1/Sp3 and AP-1 were efficiently competed by increasing molar excesses of unlabeled CLU-203/-153 (middle panels in Figure 6A,B for Sp1/Sp3 and AP-1-labeled probes, respectively). As observed in Figure 5D, the CLU/mutant 2 unlabeled oligomer was inefficient at competing the Sp1/Sp3 complex formation in the control condition (Ctrl) (lanes 2 to 7, right panel in Figure 6A), whereas it was very effective at competing for the formation of the AP-1 complex (lanes 2 to 7, right panel in Figure 6B). Interestingly, in each acquisition with the Sp1/Sp3-labeled probe (Figure 6A), we observed a stronger, more intense signal for the DNA-protein complexes in the control (Ctrl) than in the injured condition (Wounded). Indeed, the addition of a 500-fold molar excess of the unlabeled Sp1/Sp3 (left panel, Figure 6A) or CLU-203/-153 oligomers (middle panel, Figure 6A) entirely prevented the formation of the TFs complexes in the wounded but not the control condition (compare lane 7 and 13). In contrast, the AP-1-labeled probe clearly yielded weaker formation of the AP-1 complexes in the control condition compared to damaged cells (Figure 6B). Indeed, the addition of a 500-fold molar excess of either unlabeled AP-1 (left panel) or CLU-203/-153 (middle panel) completely inhibited formation of the AP-1 complex in the control condition, whereas it is still present (although reduced) in the wounded condition (compare lane 7 and 13). As the CLU/mutant 2 oligomer bears a mutated Sp1/Sp3 site but has an intact AP-1 site, it competed (right panel) as efficiently as the high affinity AP-1 competitor (left panel) for the binding of this TF. In addition, one can clearly appreciate the much stronger binding of AP-1 to the labeled probe when equal amounts of nuclear proteins from wounded hCECs are used relative to those from control cells (compare lanes 2 to 7 with lanes 8 to 13).

We next monitored the expression of both Sp1/Sp3 and AP-1 at the protein level by Western blot in nuclear extracts prepared from both unwounded and scratch-wounded hCECs using antibodies specific to both Sp1 and Sp3, and the c-Fos, c-Jun and b-Jun AP-1 isoforms. As shown in Figure 3B, the mRNA transcripts encoding all these TFs are expressed in hCECs. Surprinsingly, although c-Fos, c-Jun and b-Jun mRNA expression is increased after hTECs injury (Figure 3B), expression of these proteins is, on the other hand, reduced under this cell culture condition, therefore suggesting that the steady-state level of the AP-1 isoforms is likely subjected to post-translational regulation (Figure 6C). On the other hand, and as observed for their corresponding mRNA transcripts, expression of both the Sp1 and Sp3 proteins is reduced in wounded hCECs (Figure 3B).

## 3. Discussion

Due to its anatomical localization, the cornea is the first structure of the eye to be altered by external (chemical, thermal, biological or physical) aggressions. In the most severe cases, a medical treatment with anti-inflammatory and/or pro-healing substances is not sufficient and corneal transplantation or eye enucleation may even be required. In 2012, it was estimated that 12.7 million people were waiting for a corneal transplantation worldwide. Unfortunately, only 185,000 corneal transplantations could be performed in the world that same year, covering the needs of a mere 1 in 70 patients [58]. Therefore, in order to reduce the needs for donor corneas, the use of an entirely tissue-engineered human cornea (hTECs) that can be used as a model to study the mechanisms of wound healing is of prime importance, as it may help develop alternative solutions to the need of graftable, donor corneas. Over the last 20 years, we succeeded in producing a human tissue-engineering corneal substitute (hTEC), which, besides being devoid of any synthetic materials, exhibits a well-developed stratified epithelium made up of 5–7 layers of untransformed human corneal epithelial cells (hCECs) and a stroma. Because of these similarities with the native cornea, our hTEC represents an outstanding model that we can exploit to study in detail the cellular and molecular mechanisms of corneal wound healing as a prerequisite to further studies in animals [9,11].

In the present study, we identified the *CLU* gene as among the most severely deregulated genes during wound healing of hTECs. Western blot analyses carried out both on hCECs (2D model) and hTECs (3D model) present similar trends, with the reduction being more significant in the hTECs. Considering the predominant role of the ECM during corneal repair and that some links have been established between CLU and ECM components [59,60,61], these results are not that surprising. CLU involvement in cell adhesion, migration and proliferation is now broadly accepted, but the molecular basis of its regulation remains elusive. Indeed, CLU negatively regulates fibronectin and type I collagen expression [59]. Therefore, its down-regulation following hTEC injury is consistent with the well-known fibronectin increase that occurs early during the healing process [62,63]. MMP expression is also profoundly altered in response to the changes in the ECM composition (such as a reduction of fibronectin) that occurs during corneal wound healing [64]. Our gene-profiling analyses revealed that MMP-9 and MMP-10 are among the 54 most differentially expressed genes with a robust increase of their expression in the wounded condition (Figure 1B). Interestingly, clusterin has been reported to interact with MMP-9 to inhibit MMP-9-mediated breakdown of the tight junctions between human epithelial cells [60]. Therefore, the decreased clusterin expression observed in the wounded area correlates with the increased expression of MMP-9 and MMP-10 (Figure 1B), which is also consistent with both the proper ECM remodeling and cell migration that takes place in order to regenerate the corneal layer. On the other hand, collagen facilitates CLU gene expression [65]. Again, the reduction of collagen expression that typically occurs early during corneal wound healing [62] is consistent with the decreased expression of clusterin, as demonstrated in our study, and suggests a possible regulatory feedback loop between ECM components and CLU expression.

Transfection analyses revealed that one positive and two negative (a strong and a moderate one) regulatory regions are present along the *CLU* gene promoter and 5′-flanking region (Figure 2C). Among reports identifying TF binding sites along the *CLU* gene promoter [37,38], only a few characterized the regulatory sequences needed to ensure proper transcription of the *CLU* gene [39,40,41,42,43,44,45,46]. However, none of them have been done in the context of human wound healing of the cornea. The proximal element identified in our study (-82/-203) bears target sites for TBP (TATA-binding protein), AP-1 and both the Sp1 and Sp3 family members (Figure 3A), which we are very familiar with [11,17,18,53,66]. We demonstrated that *CLU* gene transcription was indeed ensured in part by the binding of the TFs Sp1/Sp3 and AP-1 to overlapping target sites within the basal promoter segment CLU -203/-153. While AP-1 has a stronger regulatory influence than Sp1/Sp3, these latter, however, have a clearly greater affinity for their target site than AP-1. Interestingly, both Sp1/Sp3 expression (Figure 6C) and DNA binding (Figure 6A) to the *CLU* basal promoter were decreased in context of injury. This result is consistent with the regulation of *CLU* expression observed on microarrays (Figure 1B) and western blot analysis (Figure 1C) during wound healing when one considers that Sp1/Sp3 is a well-known transcriptional activator. The fact that TFs expression was evaluated in nuclear extracts obtained from hCECs and not in hTECs must be taken into consideration. According to the previous observations that showed a more robust *CLU* regulation after wounding in hTECs compared to hCECs, it is, therefore, not surprising that the impact of Sp1/Sp3, but also AP-1, on *CLU* gene expression was also relatively moderate in hCECs. Our results demonstrated that AP-1 binds to the CLU -203/-153 region (Figure 6B) without distinguishing the AP-1 subunits involved in this complex formation. Surprisingly, while microarray (Figure 3B) and EMSA analysis revealed a greater binding of the AP-1 subunit on DNA in wounded cells (Figure 6B), protein analysis revealed a decrease in c-Fos, b-Jun and c-Jun expression (Figure 6C), which can be explained by three mains hypotheses. First, Jun proteins can associate between each other to form homo- and hetero-dimers, whereas Fos proteins can only heterodimerize. Heterodimers, such as c-Jun/c-Fos, are more stable and have a greater transcriptional activity [67,68]. Thus, the dimerization partners of Jun subunits impact on its role in gene activation and cell cycle regulation [69,70]. Jun and Fos families positively regulate cell proliferation, whereas b-Jun inhibits proliferation. This effect of b-Jun is probably mediated by its ability to dimerize with c-Fos, the b-Jun/c-Fos heterodimer often being reported to repress gene transcription [71,72,73]. According to our gene profiling analysis (Figure 3B), important changes in the pattern of AP-1 subunit gene expression occurs between unwounded and wounded hTECs. Indeed, expression of *JUN* (c-Jun), *FOS* (c-Fos) but most particularly *JUNB* (b-Jun) is considerably increased in the context of wound healing. The EMSA analysis also revealed an increase of AP-1 complexes formation in the context of an injury (Figure 6B). Therefore, it is most likely that wounded and unwounded hTECs could express AP-1 proteins made up of different constituting subunits that then reorganized (with the disappearance of b-Jun, c-Jun and c-Fos subunits heterodimers) upon complete closure of the wounds. Regarding their function, this imbalance during wound healing could reduce the transactivator potential of AP-1, which is consistent with the decrease in the *CLU* gene expression we observed.

The fact that expression of the AP-1 subunits c-Fos, b-Jun and c-Jun at the protein level (all reduced in wounded hCECs) does not correlate with their corresponding mRNA expression at the transcriptional level (which are abundantly expressed in hTECs) is not unusual, as such discrepancies between expression of a specific gene at the mRNA and protein levels are common. It is well-known that AP-1 TFs are subjected to a variety of post-translational modifications, such as protein phosphorylation, glycosylation or ubiquitinylation, which can alter their activity, stability, localization and DNA-binding properties [74,75,76,77,78,79], and may, therefore, contribute to the differences observed in wounded cells (Figure 6B). C-Fos, c-Jun and FOSL2 have all been reported to be degraded by the proteasome [78,79]. Interestingly, c-Fos is degraded by the proteasome independently of its own ubiquitination level [75], whereas c-Jun is only degraded in an ubiquitin-dependent manner [77]. Recent evidences also suggest that the c-Jun/c-Fos dimer can be degraded through the SUMO pathway [76]. Alternatively, it is well-known that the protein product of some genes negatively regulates their own transcription through a negative feedback loop mechanism that often involves a specific microRNA (miRNA) [80].

Finally, we demonstrated that the CLU -203/-153 promoter segment preferentially binds Sp1/Sp3 rather than AP-1. In the context of healing, Sp1/Sp3 is decreased while AP-1 is increased (Figure 6A,B). These results can be the consequence of modifications in the TFs affinity for the *CLU* promoter and can be explained by the expression of a wound healing-dependent AP-1 co-activator that would facilitate the recruitment of AP-1 to the *CLU* promoter. Indeed, AP-1-dependent gene expression is regulated by a combination of various AP-1 activators, general or basal transcription initiation factors and associated cofactors. Basal transcription by RNA polymerase II requires at least six general transcription initiation factors: TFIIA (Transcription Factor IIA), TFIIB, TFIID, TFIIE, TFIIF and TFIIH. AP-1, in particular its c-Fos-constituting isoform, has been shown to interact with both TBP (TATA binding protein) and TFIIB [81]. However, this transcriptional activation is relatively weak and needs additional transcriptional coactivators to be enhanced. Three types of proteins are among the best characterized AP-1 co-activators: CBP (CREB-binding protein), JAB1 (Jun activation domain-binding protein 1) and alpha-NAC (Nascent polypeptide associated complex And Coactivator alpha). Functional assays using transient transfection confirmed that CBP stimulates the activity of c-Jun, b-Jun and v-Jun in vivo [82], but also c-Fos [83]. Although Jun coactivation by CBP requires phosphorylation events [84], interaction of JAB1 with Jun does not require phosphorylation but remains subunit-dependent [85]. Alpha-NAC was also shown to potentiate the activity of the homodimeric c-Jun activator, while transcription mediated by the c-Fos/c-Jun heterodimer was unaffected [86]. More recently, a few more studies further identified novel AP-1 co-activators. For instance, the dimeric RACO-1 protein was identified as a c-Jun coactivator, and both YAP (Yes-associated protein) and TAZ (Transcriptional activator with PDZ domain) proteins as b-Jun co-activators required for transcriptional activation by AP-1 [87,88]. As both YAP and TAZ were recently found to be key mediators of the wound healing and tissue regeneration responses to tissue damage [89], it would prove particularly interesting to determine whether both these proteins are expressed in the hTEC and if their expression is affected by wound healing in this biological model.

Most DNA target sites identified in the distal silencer (-1424/-2000) by the TFSEARCH analysis have been reported to bind TFs such as AREB6, ARP-1, Bach1 and Evi1 (Supplementary Table S2), which have been reported to function as transcriptional repressors [90,91,92,93,94,95]. Therefore, the repressive action of these TFs, once they have bound to their target sites in the CLU silencer, may, at least in part, contribute to the differential expression observed for the *CLU* gene in our hTEC model. To support this idea, we searched our microarray data files to define whether these TFs are expressed in our tissue-engineered model. Although each of them is expressed in our hTECs, no significant variation in the expression of these TFs between unwounded and wounded hTECs was observed (Supplementary Figure S1). Further studies will be required in order to demonstrate the binding of some of these negative TFs to the *CLU* distal silencer and whether they indeed contribute to the repression of *CLU* gene expression during hTEC wound healing.

## 4. Materials and Methods

This study was conducted in accordance with our institution’s guidelines and the Declaration of Helsinki. The protocols were approved by the CHU de Québec—Université Laval hospital and Université Laval Committee for the Protection of Human Subjects (DR-002-955 / Modification F1-47318).

### 4.1. Cell Culture—Human Epithelial Cells (hCECs)

Human corneal epithelial cells (hCECs) were isolated from the limbal area of normal eyes (obtained from the Banque d’Yeux Nationale of the Centre Universitaire d’Ophtalmologie; CHU de Québec, Hôpital du Saint-Sacrement, Québec, QC, Canada) of 52-, 70-, 73- and 74-year-old donors as previously reported [9,16,55]. hCECs (seeded at 5 × 10^5^ cells/T75 flask) were cultured with a feeder layer of irradiated human fibroblasts (iHFLs). iHFLs were isolated from the foreskin of a 10-day-old donor and cultured in DH medium (Dulbecco-Vogt modification of Eagle’s medium with Ham’s F12 at a 3:1 ratio) with supplements (5% Fetal Clone II serum, 5 μg/mL insulin, 0.4 μg/mL hydrocortisone, 10 ng/mL epidermal growth factor, 10^−10^ mol/L isoproterenol, 100 μg/mL Penicilin, and 25 μg/mL Gentamycin) at 4.5 × 10^5^ cells/T75 flask, as recently described [96]. All cells were grown under 8% CO_2_ at 37 °C and culture medium was changed three times a week [9]. At each passage, cell number was counted with a Beckman Coulter. hCEC-52, hCEC-70X, hCEC-73X and hCEC-74Y (5 × 10^5^ cells) were each plated in duplicates (for total proteins extraction) or quadruplicate (for nuclear proteins extraction) with iHFLs (4.5 × 10^5^ cells) in 100 cm^2^ tissue-culture dishes in complete DH medium. When cells reached confluence, eight scratches (four horizontal and four vertical) were created in the center of the plate using a P1000 pipet tip (Sarstedt, Nümbrecht, Germany). Wound closure was monitored and cells were then harvested to proceed immediately to extraction of the nuclear proteins.

### 4.2. Human Tissue-Engineered Cornea (hTEC)

hTECs were produced according to the self-assembly approach [9,13,16]. Human corneal fibroblasts were isolated from the stromal portion of a cornea (from a 26-day-old donor), left after dispase digestion and removal of both the endothelium and epithelium and primary cultured and sub-cultured as previously reported [9,16]. Human skin fibroblasts were obtained from the dermal portion of adult breast skin and were cultured as described previously [9,97]. Briefly, corneal and dermal fibroblasts were seeded and cultured in fibroblast growth medium (Dulbecco modified Eagle medium—DMEM—supplemented with 10% fetal calf serum, 25 µg/mL Gentamycin and 100 µg/mL Penicillin) and prompted to lay down their own ECM by the further addition of 50 mg/mL ascorbic acid (Sigma, Oakville, ON, Canada) for 28–35 days, as previously described [9,13,15]. After peeling from the flasks, two tissue sheets were superimposed to form a reconstructed stroma and were cultured for another week so that they could adhere to each other. Then, human corneal epithelial cells (hCECs) were seeded on the surface of the reconstructed stroma and cultured in submerged conditions in complete epithelial cell medium (DH medium) supplemented with ascorbic acid. After 7 days, reconstructed tissues were fed an EGF-free epithelial cell medium and were raised at the air-liquid interface for 7 days to induce epithelial differentiation. Reconstructed partial thickness corneas produced by the self-assembly approach were then wounded using an 8-mm biopsy punch. After wounding, the tissue-engineered corneas were placed over two supplementary fibroblast sheets to allow reepithelialization over a natural matrix. Wound closure was then examined macroscopically every 24 h for 4 days following the initial damage by observing the ring of reepithelialization that progressed toward the wound center using a Zeiss Imager.Z2 microscope (Zeiss Canada Ltd., North York, ON, Canada) equipped with a numeric CCD camera (AxioCam MRm; Zeiss). All experiments were repeated four times. Epithelial tissues from the central area of both wounded and unwounded (used as negative controls) hTECs were harvested 4 days post-wounding to collect proteins required for western blot analyses.

### 4.3. Gene Expression Profiling

Microarray analyses were conducted by the gene profiling service of the CUO-Recherche (Québec, QC, Canada), as done previously [11]. As biological replicates, total RNA was obtained from three different populations of HCECs (44, 52 and 71 years old). Total RNA was isolated from the epithelial cells isolated from the central area of both wounded and unwounded (used as negative controls) hTECs using the RNeasy Mini Kit (QIAGEN,Toronto, ON, Canada) and its quality determined (2100 bioanalyzer, Agilent Technologies, Mississauga, ON, Canada) as recently described [11]. Because corneal fibroblasts are much less abundant (36.2+/−1.0%) than epithelial cells (63.9+/−0.9%) in the hTECs and as they are trapped in the stromal collagen matrix and not mitotically active, they will not significantly contribute to the total RNAs isolated, as nearly all of it will originate from the epithelial cells. Labeling of cyanine 3-CTP labeled targets, their hybridization on a G4851A SurePrint G3 Human GE 8 × 60Karray slide (Agilent Technologies) and data acquisition and analyses were all done as previously reported [11] (GSE #75336). All data generated from the arrays were also analyzed by robust multi-array analysis (RMA) for background correction of the raw values. They were then transformed in Log2 base and quantile normalized before a linear model was fitted to the normalized data to obtain an expression measure for each probe set on each array. Scatter plots and heat maps were generated using the ArrayStar V4.1(DNASTAR, Madison, WI, USA) software. All microarray data presented in this study comply with the Minimum Information About a Microarray Experiment (MIAME) requirements (GEO# GSE75336; https://www.ncbi.nlm.nih.gov/geo/query/acc.cgi?acc=GSE75336; last accessed date: 15 November 2021).

### 4.4. Western Blot Analyses

Extraction of proteins from both wounded and unwounded hTECs and hCECs was performed in TGNT lysis buffer and protein concentration evaluated by the Bradford procedure and further validated following Coomassie Blue staining of SDS-polyacrylamide fractionated nuclear proteins. Western blots were conducted as described [54] using the following primary antibodies: rabbit polyclonal antibodies against CLU (1:500; Santa Cruz Biotechnology, Dallas, TX, USA; detects endogenous levels of sCLU, nCLU and cCLU), Sp1 (1:250; Abcam Inc., Toronto, ON, Canada), Sp3 (1:1000; Santa Cruz Biotechnology), c-Fos (1:1000; Santa Cruz Biotechnology), b-Jun (1:200; Santa Cruz Biotechnology), c-Jun (1:1000; Santa Cruz Biotechnology), actin (1:40,000; Santa Cruz Biotechnology) and a peroxidase-conjugated AffiniPure Goat secondary antibody against mouse and rabbit IgG (1:2500; Jackson Immuno Research Laboratories, Baltimore, PA, USA). Blots on CLU were realized using total proteins while those for the evaluation of transcription factors (Sp1, Sp3, cFos, b-Jun and cJun) were obtained with the same nuclear extracts as those used for EMSA. The labeling was revealed using a ECL Detection Kit (Amersham, Baie d’Urfé, QC, Canada) as described [52,57]. Measurement of the densitometric intensity of the protein bands was determined and normalized to that of the corresponding actin band with the Image Studio Lite software (Image Studio Lite version 5.2.5, LI-COR Biosciences, Lincoln, NE, USA).

### 4.5. Plasmid Constructs and Oligonucleotides

The details regarding the production of the different recombinant plasmids used in this study are provided in the Supplementary Materials. The double-stranded oligonucleotides used either as labeled probes or unlabeled competitors in the EMSAs were chemically synthesized using a Biosearch 8700 apparatus (Integrated DNA Technologies, Inc., Coralville, WA, USA). Their DNA sequences are listed in Supplementary Table S1.

### 4.6. Transient Transfection and CAT (Chloramphenicol Acetyl Transferase) Assays

All CLU/CAT recombinant plasmids were transiently transfected into primary cultured hCECs (hCEC-52, hCEC-70X and hCEC-73X plated in triplicates) grown to sub-confluence (70% coverage of the culture plate) into 6-well tissue-cultured plates using the Viafect Transfection Regent (Promega North America, Madison, WI, USA). Each tissue-cultured well received 1.5 μg of the test plasmid and 0.5 μg of the hGH-encoding plasmid PXGH5. All cells were harvested 48 h following transfection and CAT activities were determined and normalized to both the hGH secreted in the medium and total proteins, as previously described [98].

### 4.7. Preparation of Nuclear Extracts and Electrophoretic Mobility Shift Assays (EMSA)

hCECs (hCEC-52, hCEC-70X, hCEC-73X) at passages 3 to 4 were seeded in the presence of iHFL at 5 × 10^5^ cells/cm^2^ in 100 cm^2^ tissue-culture dishes and grown for four days at 37 °C. When cells reached near-confluence (which corresponds to 90% coverage of the culture dishes), 10 scratches (five horizontal and five vertical) were created using a P1000 pipet tip (Sartsted, Nümbrecht, Germany). As a negative control, hCECs at the same passages were also seeded without scratches. Nuclear extracts were then prepared from all cultured cells, dialyzed and kept frozen in small aliquots at −80 °C until they were used for EMSAs as previously described [56,99]. Briefly, EMSAs were carried out by incubating nuclear proteins with either a 5′^32^P-end-labeled, 50 bp DNA fragment bearing most of the *CLU* proximal activator region from position -203 (SacII site) to -153 relative to the *CLU* mRNA start site and designated CLU-203/-153, or a 5′^32^P-end-labeled oligomers bearing the high affinity recognition sequence for the transcription factors AP-1 or Sp1/Sp3. Approximately 8 × 10^4^ cpm labeled probe was incubated with 15 µg nuclear proteins in the presence of 0.5 μg of poly(dI:dC) (AmershamBiosciences, Piscataway, NJ, USA) and 50 mM KCl in buffer D [10 mM Hepes pH 7.9, 10% *v*/*v* glycerol, 0.1 mM EDTA, 0.5 mM DTT (dithiothreitol; Sigma-Aldrich Canada, Oakville, ON, Canada) and 0.25 mM phenylmethylsulfonyl fluoride (PMSF; Sigma-Aldrich Canada)]. DNA–protein complexes were next separated by gel electrophoresis through 6% non-denaturing, native polyacrylamide gels run against Tris-glycine buffer (50 mM Tris, 2.5 mM EDTA, 0.4 M glycine) at 120 V or 110 V (as specified in the Figure legends) for 6.5 h, 4 °C. The position of the DNA–protein complexes was then revealed upon autoradiography of the dried gels at −80 °C. When specified in the legend of each figure, synthetic double-stranded oligonucleotides bearing the recognition sequence for known transcription factors (AP-1 or Sp1/Sp3), or that of either the wild type or mutated CLU-203/-153 50 bp DNA fragment (Supplementary Table S1), were added as unlabeled competitors (150- to 500-fold molar excesses) during the assay. Super-shift experiments were conducted by incubating 15 μg nuclear proteins from hCECs in the presence of 1 µg of a polyclonal antibody raised against AP-1 (cFos–sc 166,940 and cJun–sc44X, Santa Cruz Biotechnology, Dallas, TX, USA), Sp1 (Ab13370–Abcam Inc.) or Sp3 (sc-644; Santa Cruz Biotechnology, Dallas, TX, USA).

### 4.8. Statistical Analyses

Student’s *t*-test was performed for comparison of the groups for quantification of the western blot signals shown in Figure 1D and Figure 6C. Differences were considered to be statistically significant at *p* < 0.05. After a first *t*-test analysis, and when this one is significant, a comparison of means was also performed by an ANOVA test (Tukey) using GraphPad PRISM software (GraphPad Software Inc., La Jolla, CA, USA) for the analysis of the transfection data presented in Figure 2B. All data are also expressed as mean ± SD.

## 5. Conclusions

In this study, we demonstrated that the expression of clusterin is dramatically repressed (at both the gene and protein levels) during closure of hTEC wounds. This reduced expression of CLU gene transcription was found to be mediated, at least in part, by alteration in both the expression and DNA binding of the transcription factors AP-1 and Sp1/Sp3, which we demonstrated to bind overlapping target sites in the basal promoter of the human CLU gene.

## Data Availability

All microarray data presented in this study can be accessed at NCBI Gene Expression Omnibus (GEO# GSE75336; https://www.ncbi.nlm.nih.gov/geo/query/acc.cgi?acc=GSE75336, last accessed date: 15 November 2021).

## Supplementary Materials

The following are available online at https://www.mdpi.com/article/10.3390/ijms222212426/s1.
