# Supplementary material for: Contribution of the Transcription Factors Sp1/Sp3 and AP-1 to Clusterin Gene Expression during Corneal Wound Healing of Tissue-Engineered Human Corneas
Source: Int J Mol Sci. 2021 Nov 17;22(22):12426. doi: 10.3390/ijms222212426 (PMC8621254; doi:10.3390/ijms222212426)
Supplement: Supplementary file 1 [file ijms-22-12426-s001.zip › ijms-1430236-supplementary.pdf]

SUPPLEMENTARY MATERIAL

Supplementary Methods

Plasmid constructs

A 2 kb fragment bearing the 5′ flanking sequence of the human CLU gene (from position -2000 to +1 relative to the theoretical mRNA start site) was synthesized and cloned by TOP Gene Technologies Inc. (St-Laurent, Québec, Canada) upstream of the CAT reporter gene into the pCATBasic vector (Promega, Madison, WI, USA). Derivatives from the -2000/CLU construct bearing various deletions of the CLU promoter were then produced by single restriction enzyme digestion of the parental plasmid. Each restriction enzyme has a first recognition site located 5′ into the multiple cloning site (MCS) of pCATBasic and cuts a second time in the -2000/CLU sequence (BssHII (cuts at position -82), SacII (cuts at -203), NotI (cuts at -334), SwaI (cuts at -503), BstXI (cuts at -748), BglII (cuts at -917), AvrII (cuts at -1424) and SacI (cuts at -1737)). The restriction site overhangs of the double-digested plasmids were then ligated using T4 DNA ligase (New England Biolabs). All recombinant CLU/CAT plasmids therefore share the same 3′ end (at position +82) but different 5′ termini (5′ positions: -2000, -1737, -1424, -917, -748, -503, -334 -203, -82).

Supplementary tables

Supplementary Table S1. Oligonucleotides used as labeled probes or competitors in the EMSAs

**Oligonucleotide**	**Top strand (5′-3′)**<br>**Bottom strand (5′-3′)**
AP1	GATCCCCGCGTTGAGTCATTCGCCTC<br>GATCGAGGCGAATGACTCAACGCGGG
Sp1/3	GATCATATCTGCGGGGCGGGGCAGAC<br>GATCCTGTGTCTGCCCCGCCCCGCAG
NFI	TTATTTTGGATTGAAGCCAATATGAG<br>CTCATATTGGCTTCAATCCAAAATAA
CLU -203/-153	GATCCGGCATTCTTTGGGCGTGAGTCATGCAGGTTTGCAGCCAGCCCCAAAGGGG<br>GATCCCCCCTTTGGGGCTGGCTGCAAACCTGCATGACTCACGCCCAAAGAATGCC
CLU -203/-153 Mutant 1	GATCCGGCATTCTTTGGGCGTGAGTCATGCAGGTTTGCA**TTT**AG**TTT**CAAAGGGG<br>GATCCCCCCTTTG**AAA**CT**AAA**TGCAAACCTGCATGACTCACGCCCAAAGAATGCC
CLU -203/-153 Mutant 2	GATCCGGCATTCTTT**TTTTT**TGAGTCATGCAGGTTTGCAGCCAGCCCCAAAGGGG<br>GATCCCCCCTTTGGGGCTGGCTGCAAACCTGCATGACTCA**AAAAA**AAAGAATGCC

Supplementary Table S2. TFSEARCH analysis on the CLU -1424/-2000 promoter segment

seq_CLU -1424/-2000

Name	Sequence	Position	Strand	Score	p-value	E-value
FOXJ2(M00422)	TACTAAGCAAACAAGGTT	-1992	-	9.42	0.000275	0.068
HNF-3beta(M00131)	CCTTGTTTGCTTA	-1990	+	16.94	0	0
HFH-8(M00294)	CCTTGTTTGCTTA	-1990	+	9.57	0.000325	0.082
HFH-3(M00289)	CCTTGTTTGCTTA	-1990	+	10.5	0.000175	0.044
FOXD3(M00130)	CCTTGTTTGCTT	-1990	+	8.09	0.000475	0.120
Ik-3(M00088)	TAGTGGGAATAAT	-1976	+	10.81	0.0001	0.0251
Ik-1(M00086)	TAGTGGGAATAAT	-1976	+	9.95	0.00015	0.038
AhR:Arnt(M00237)	AATAATTACTTGATTATTC	-1970	-	1	0.000975	0.239
Nkx2-5(M00240)	TCAAGTA	-1964	+	9.02	0.000875	0.225
Olf-1(M00261)	AATTATTCCCAAGGGATCACAG	-1958	+	9.57	0.000225	0.054
Ik-3(M00088)	CCTTGGGAATAAT	-1957	-	6.92	0.00095	0.238
Ik-1(M00086)	CCTTGGGAATAAT	-1957	-	12.55	0	0
Ik-1(M00086)	CCTTGGGAATAA	-1956	-	8.32	0.000625	0.157
Ik-2(M00087)	CCTTGGGAATAA	-1956	-	10.77	0.000125	0.0315
ARP-1(M00155)	TGATCCCTTGGGAATA	-1955	-	10.15	5.0E-5	0.0124
Poly A downstream element(M00211)	TGTGATCCC	-1946	-	9.92	7.5E-5	0.0191
SEF-1(M00214)	CACAGTCATGACTGTGATC	-1944	-	6.51	0.000325	0.080
TCF11:MafG(M00284)	CAGTCATGACTGTGAATCTGT	-1939	+	11.59	0.000625	0.151
Pax-4(M00380)	AAAATAGCATGATTCACAGATTCACAGT	-1931	-	18.34	0.000125	0.0293
Pax-4(M00380)	AGAAAATAGCATGATTCACAGATTCACAG	-1930	-	16.06	0.000825	0.193
MEIS1B:HOXA9(M00421)	TCACAGATTCACAG	-1930	-	9.02	0.000425	0.106
AREB6(M00414)	GTGAATCTGTGA	-1928	+	7.69	0.00095	0.239
ARP-1(M00155)	TGAATCTGTGAATCAT	-1927	+	6.3	0.000825	0.205
Bach1(M00495)	TCTGTGAATCATGCT	-1923	+	8.64	0.000175	0.044
Bach1(M00495)	CTGTGAATCATGCT	-1922	+	5.55	0.00085	0.212
AP-1(M00517)	GCATGATTCACAG	-1922	-	8.56	0.000575	0.144
AP-1(M00199)	GTGAATCA	-1920	+	12.15	0.000525	0.134
Xvent-1(M00445)	ATGCTATTTTC	-1913	+	13.49	0.000875	0.220
Lyf-1(M00141)	CTTGGGAGA	-1904	-	9.63	0.0001	0.0255
Nkx2-2(M00485)	CAAGTCCTT	-1899	+	15.77	0	0
STATx(M00223)	TTCCCCTAA	-1875	+	10.58	0.000175	0.045
MyoD(M00184)	TTCAGCTGGC	-1866	+	8.13	0.0007	0.178
AP-4(M00176)	TTCAGCTGGC	-1866	+	8.52	0.000575	0.146
AP-4(M00176)	TCAGCTGGC	-1865	+	8.52	0.000575	0.146
CP2(M00072)	GCCTTAGCCAG	-1861	-	8.21	0.0007	0.177
GC box(M00255)	CTAAGGCGGATCTC	-1857	+	9.19	0.00025	0.063

Sp1(M00196)	CTAAGGCGGATCT	-1857	+	8.39	0.000275	0.069
Elk-1(M00007)	TAAGGCGGATCTCACT	-1856	+	8.73	0.00035	0.087
AP-2rep(M00468)	CAGTGAG	-1846	-	6.45	0.00095	0.244
AP-1(M00517)	AGGTCAGTCAGTG	-1844	-	7.73	0.00095	0.238
NF-E2(M00037)	CACTGACTGAC	-1844	+	8.27	0.000775	0.196
AP-1(M00172)	ACTGACTGACC	-1843	+	8.57	0.0009	0.228
AP-1(M00174)	ACTGACTGACC	-1843	+	9.34	0.000425	0.108
AP-1(M00173)	ACTGACTGACC	-1843	+	8.46	0.000925	0.234
AP-1(M00188)	ACTGACTGACC	-1843	+	10.35	0.0001	0.0253
RORalpha1(M00156)	CTTTGGAGGTCA	-1837	-	15.01	0.0001	0.0251
Evi-1(M00081)	AAATATAACTAGATT	-1813	+	7.83	0.00075	0.187
Freac-7(M00293)	AAGGCATAAATCTAGT	-1806	-	8.35	0.00085	0.211
TBP(M00471)	CATAAATC	-1802	-	8.2	0	0
Sp1(M00008)	AAGGCATAAA	-1800	-	7.72	0.0002	0.051
Tal-1beta:ITF-2(M00070)	CGGAACAGAAGGCATA	-1798	-	7.83	0.0002	0.050
NRSF(M00256)	CTCAGCATCCGGAACAGAAGG	-1794	-	-1.73	0.000725	0.176
NRSF(M00256)	CTCAGCATCCGGAACAGAAG	-1793	-	-1.73	0.000725	0.176
Elk-1(M00025)	TGTTCCGGATGCTG	-1789	+	9.78	0.000275	0.069
AP-2(M00189)	GTTCCGGATGCT	-1788	+	8.07	0.00045	0.113
c-Ets-1(p54)(M00074)	TTCCGGATGCTG	-1787	+	12.8	0.00095	0.238
c-Ets-1(p54)(M00074)	TCCGGATGCTG	-1786	+	13.52	0.000275	0.069
RP58(M00532)	TCAGCATCCGGA	-1786	-	5.26	0.000675	0.170
c-Ets-1(p54)(M00032)	TCCGGATGCT	-1786	+	11.57	5.0E-5	0.0127
c-Ets-1(p54)(M00074)	CCGGATGCTG	-1785	+	13.92	0.000125	0.0314
NRSF(M00256)	TTCTGTAGCTCCCTCAGCATC	-1782	-	-0.63	0.00045	0.109
myogenin / NF-1(M00056)	AACTTTTCTGTTCTGTAGCTCCCTCAGCA	-1780	-	6.45	0.000675	0.159
E2(M00107)	AGAACAGAAAAGTTAC	-1765	+	7.54	0.00095	0.236
VBP(M00228)	GTTACA	-1754	+	15.31	0.000675	0.171
Sox-5(M00042)	AACAATTC	-1733	+	12.75	0.00085	0.258
MyoD(M00001)	CCTCAGGTATTG	-1719	+	8.96	0.000425	0.128
SOX-9(M00410)	CCTTATCAATACCT	-1715	-	8.17	0.0008	0.240
GATA-2(M00348)	ATTGATAAGG	-1711	+	9.25	0.0005	0.152
GATA-1(M00347)	ATTGATAAGG	-1711	+	10.02	0.000225	0.068
Lmo2 complex(M00278)	TGATAAGG	-1709	+	11.88	0.000875	0.267
GATA-6(M00462)	ACTGATAAGG	-1694	+	8.18	0.000425	0.129
GATA-2(M00348)	ACTGATAAGG	-1694	+	10.37	2.5E-5	0.0076
GATA-1(M00347)	ACTGATAAGG	-1694	+	9.9	0.00025	0.076
GATA-1(M00346)	ACTGATAAGG	-1694	+	9.5	0.000275	0.084
GATA-1(M00128)	ACTGATAAGGAG	-1694	+	14.32	0.000125	0.038

GATA-3(M00077)	CTGATAAGG	-1693	+	7.72	0.000975	0.297
Lmo2 complex(M00278)	TGATAAGG	-1692	+	11.88	0.000875	0.267
GR(M00192)	GGAGCAGGCACTGTCCACA	-1686	+	9.6	0.000275	0.081
Sp1(M00008)	CAGGCACTGT	-1682	+	6.89	0.0008	0.243
C/EBP(M00159)	TCTGTGGACAGTG	-1678	-	7.71	0.000875	0.263
Msx-1(M00394)	CAGAAAATG	-1669	+	7.24	0.0007	0.213
ISRE(M00258)	CAGCTACATTTTCTG	-1669	-	6.2	0.001	0.299
BSAP(M00143)	TTTAAGAAAAATAAAGTGTAGCCTCTTC	-1630	+	7.44	0.00085	0.243
Olf-1(M00261)	AAAATGTCCCAGAAGAGGCTAC	-1613	-	12.27	5.0E-5	0.0146
Olf-1(M00261)	AAAATGTCCCAGAAGAGGCTA	-1612	-	9.21	0.00035	0.102
STATx(M00223)	TCCCAGAA	-1605	-	7.55	0.000825	0.252
GR(M00192)	GAAGAAAAAAATGTCCCAG	-1603	-	10.61	0.0001	0.0295
SRY(M00148)	AATGTCC	-1600	-	6.04	0.0001	0.0307
YY1(M00069)	AGAAGGCCATTAACCCAGAA	-1584	-	8.38	0.000675	0.198
PPAR(M00528)	TTCTGGGTTAATGGCCT	-1584	+	10.84	0.000125	0.037
YY1(M00059)	GAAGGCCATTAACCCAG	-1582	-	8.76	0.00035	0.104
NF-1(M00193)	ACTTGGCTTGGCTCTAGG	-1561	+	9.07	0.000475	0.141
NF-1(M00193)	GCTTGGCTCTAGGTTAGG	-1556	+	8.33	0.00075	0.222
ZID(M00085)	TGGCTCTAGGTTA	-1553	+	12.07	2.5E-5	0.0075
ZID(M00085)	GGCTCTAGGTTA	-1552	+	6.66	0.000875	0.263
STAT1(M00224)	GATCCATTCCCG	-1518	+	11.73	0.000625	0.183
Ik-1(M00086)	AATCGGGAATGGA	-1516	-	8.25	0.00065	0.196
GATA-6(M00462)	CCCGATTCCT	-1510	+	8.31	0.00025	0.076
GATA-1(M00075)	CCCGATTC	-1510	+	12.53	0.000475	0.144
AP-1(M00517)	CGATTCCTCATCG	-1508	+	8.19	0.000725	0.218
Tal-1beta:E47(M00065)	TCGTCCAGATGGAAGA	-1498	+	8.71	0.000225	0.067
GATA-1(M00075)	CCAGATGG	-1494	+	12.03	0.001	0.304
NF-E2(M00037)	AACTGAGGCCC	-1482	+	9.12	0.000525	0.159
PPARG(M00512)	ACTGAGGCCCAAGGGCA	-1481	+	8.24	0.000525	0.154
GCNF(M00526)	CTGAGGCCCAAGGGCAAA	-1480	+	3.29	0.000775	0.229
HNF-4alpha1(M00411)	GAGGCCCAAGGGCA	-1478	+	10.39	0.000525	0.157
PPARG(M00515)	CACTGGGTGACCTCGGACTA	-1457	-	8.74	0.000925	0.269
RORalpha1(M00156)	AGTCCGAGGTCA	-1456	+	13.66	0.000225	0.068
v-ErbA(M00239)	GTCCGAGGTCACCCAG	-1455	+	9.3	0.0004	0.119
CREB(M00039)	TGACCTCG	-1452	-	7.68	0.000925	0.283
Zic3(M00450)	TGGGTGACC	-1449	-	7.55	0.000275	0.084
MZF1(M00084)	TGTCTAGGGGCAC	-1439	+	12.42	0	0
MZF1(M00084)	GTCTAGGGGCAC	-1438	+	7.3	0.000775	0.233

Supplementary Figure S1. Expression of transcription factors identified in the CLU -1424/-2000 distal silencer

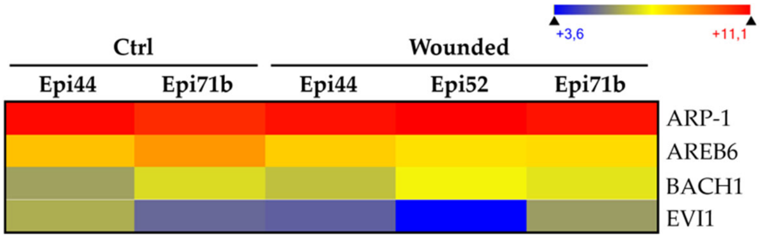

Heatmap representation of the transcriptional profiles of the TFs ARP-1, AREB6, BACH1 and EVI1 identified in the TFSEARCH analysis realized on the CLU -1424/-2000 segment, in centrally wounded relative to unwounded (negative control; Ctrl) hTECs. The color scale used to display the log2 expression level values is determined by the Hierarchical clustering algorithm of the Euclidian metric distance between genes. Genes indicated in dark blue correspond to those whose expression is very low whereas highly expressed genes are shown in orange/red.
